# Impact assessment of silver nanoparticles on plant growth and soil bacterial diversity

**DOI:** 10.1007/s13205-016-0567-7

**Published:** 2016-11-28

**Authors:** Pallavi, C. M. Mehta, Rashmi Srivastava, Sandeep Arora, A. K. Sharma

**Affiliations:** 1 Department of Biological Sciences, College of Basic Sciences and Humanities, Govind Ballabh Pant University of Agriculture and Technology, U. S. Nagar, Pantnagar, 263145 Uttarakhand India; 2 Department of Molecular Biology and Genetic Engineering, College of Basic Sciences and Humanities, Govind Ballabh Pant University of Agriculture and Technology, U. S. Nagar, Pantnagar, 263145 Uttarakhand India; 3 School of Agriculture, Lovely Professional University, Jalandhar-Delhi G.T. Road, National Highway 1, Phagwara, 144411 Punjab India

**Keywords:** Nanotechnology, Silver nanoparticles, Plant growth, Rhizosphere, Bacterial diversity, Denaturing gradient gel electrophoresis

## Abstract

The present study was carried out to investigate the impact of silver nanoparticles (AgNPs) on the growth of three different crop species, wheat (*Triticum aestivum*, var. UP2338), cowpea (*Vigna sinensis*, var. Pusa Komal), and *Brassica* (*Brassica juncea*, var. Pusa Jai Kisan), along with their impact on the rhizospheric bacterial diversity. Three different concentrations (0, 50 and 75 ppm) of AgNPs were applied through foliar spray. After harvesting, shoot and root parameters were compared, and it was observed that wheat was relatively unaffected by all AgNP treatments. The optimum growth promotion and increased root nodulation were observed at 50 ppm treatment in cowpea, while improved shoot parameters were recorded at 75 ppm in *Brassica*. To observe the impact of AgNPs on soil bacterial community, sampling was carried out from the rhizosphere of these crops at 20 and 40 days after the spraying of AgNPS. The bacterial diversity of these samples was analyzed by both cultural and molecular techniques (denaturing gradient gel electrophoresis). It is clearly evident from the results that application of AgNPs changes the soil bacterial diversity and this is further influenced by the plant species grown in that soil. Also, the functional bacterial diversity differed with different concentrations of AgNPs.

**Electronic supplementary material:**

The online version of this article (doi:10.1007/s13205-016-0567-7) contains supplementary material, which is available to authorized users.

## Introduction

The use of nanotechnology is increasing in various fields like information technology, energy, consumer goods, medical sector, and agriculture. However, every technology comes with a price; the release of NPs to the environment remains a point of concern, mostly due to the lack of scientific knowledge related to the potential health and environmental risks associated with them. Many studies have raised the concerns on how this release would affect ecosystem health and human safety (Meng et al. 2009; Klaine et al. 2008; Colvin 2003). Unfortunately, little knowledge is available to date despite these concerns. The present study deals with the effect of release of nanoparticles on plants and soil bacterial diversity.

So far, both positive and negative effects of NPs on plants were reported. Hong et al. (2005) pointed out that 0.25% nano-TiO_2_ promoted photosynthesis and nitrogen metabolism resulting in improved spinach growth. Arora et al. (2012), while studying the impact of gold NPs on the growth profile and seed yield of *Brassica juncea* under field conditions, found that out of five different concentrations of gold NPs (0, 10, 25, 50, and 100 ppm), application of 10 ppm concentration resulted in the optimum increase in growth and seed yield of the plants. Stampoulis et al. (2009) and Hawthorne et al. (2012) exposed zucchini (*Cucurbita pepo* subspecies *pepo*) to different NPs and found plant biomass reduction when compared with unexposed or corresponding bulk material controls. Musante and White (2012) reported similar trends in NP Ag and Cu phytotoxicity for squash (*C. pepo* subspecies *ovifera*), although species-specific differences existed in the magnitude of biomass, transpiration reductions, and extent of element accumulation.

Microorganisms are key regulators of biogeochemical recycling of nutrients in the environment and assist in maintaining the overall health and function of ecosystems. Microorganisms are especially sensitive to environmental changes (Sadowsky and Schortemeyer 1997); the structure and abundance of the microorganism community may shift in response to foreign nanomaterials (Ge et al. 2011; Kumar et al. 2011; Tong et al. 2007). Many nanomaterials, such as carbon nanotubes (Kang et al. 2007; Liu et al. 2009), graphene-based nanomaterials (Hu et al. 2010), iron-based nanoparticles (Auffan et al. 2009), silver (Sondi and Salopek-Sondi 2004), and copper, zinc, and titanium oxide nanoparticles (Kasemets et al. 2009), have been reported to be toxic to pure cultures of bacteria. However, these studies were conducted in vitro and it is hard to say that NPs have the same effect in the soil as they showed in in vitro conditions. Soil is a very complex system, and physicochemical characteristics such as pH, EC, texture, and organic matter content can alter the properties of NPs introduced in it. This interaction might result in increased or decreased bioavailability and toxicity of NPs (Dimkpa 2014). Many studies support this; fullerenes exposure did not alter the structure and function of the soil microbial community as reported by Tong et al. (2007), whereas Ge et al. (2011) found that nano-TiO_2_ and nano-ZnO have negative effects on the soil bacterial communities.

There are limited and inconsistent data regarding the effect of NPs on the soil microbial community and there are no standard and proven methods for assessing their toxicity on soil microbial community. Nevertheless, various methods are used to evaluate the effect of contaminants on the soil microbial community, including viability count, molecular-based methods, carbon utilization patterns, and fatty acid methyl ester (FAME) analysis. In their efforts to check the effect of AgNPs on the microbial diversity and enzyme activity of soil, Hänsch and Emmerling (2010) found a significant decrease in microbial mass with increasing AgNPs concentration. However, no treatment effects were found for microbial biomass N, fluorimetric enzymes, and the abiotic soil parameters such as pH and soil organic C.

Kirk et al. (2004) reviewed in detail the advantages and disadvantages of each method. All methods when used alone have several limitations (Mehta et al. 2014). Thus, in the current study, culture-dependent and independent methods were used in combination for assessing the ecotoxicity. The community structure was analyzed using PCR-DGGE. The present study aimed to look for the impact of AgNPs on the growth parameters of three different crop plants, *T. aestivum* (var. UP2338), *B. juncea* (var. Pusa Jai Kisan), and *V. sinensis* (var. Pusa Komal), under greenhouse conditions and their influence on rhizospheric bacterial diversity.

## Materials and methods

### Silver nanoparticles

AgNPs formulation (9 × 10^−4^ M), which was used in the present study, was synthesized at the Department of Molecular Biology and Genetic Engineering, GB Pant University of Agriculture and Technology, Pantnagar, UK, India, through chemical reduction of silver nitrate by tri-sodium citrate salt, as described by Sileikaite et al. (2006).

### Plant growth

Greenhouse experiments were conducted with three selected species, *T. aestivum* (var. UP2338), *B. juncea* (var. Pusa Jai Kisan), and *V. sinensis* (var. Pusa Komal). They were chosen as apart from being the most commonly grown crops and also represent the two major plant systems: monocots and dicots (leguminous and non-leguminous).The growth matrix used was unsterilized soil [pH 8.3, EC (dS/m) 0.852]. After 30 days of sowing, each crop was treated with 5 ml of 50 and 75 ppm concentration of AgNPs, separately as foliar spray. Distilled water was sprayed in the control treatment. Seedlings were again treated with the respective AgNPs concentrations at 40 days after sowing as a booster dose. Each treatment was replicated three times. Plant growth parameters, namely, root and shoot length, and fresh and dry weight, were recorded after 40 days of the first application of AgNPs. In the case of cowpea, root nodules were also counted in each root and their mean was taken. Another set of experiment was performed with the same conditions, but without any crops, and AgNPs were directly added to the soil.

### Bacterial community analysis

To determine the effect of AgNPs on soil bacterial diversity, soil samples were taken from the rhizosphere of the three plant species after 20 and 40 days of the first AgNPs treatment. Then, these samples were analyzed by a culture-dependent (serial dilution plate count) method on different agar medium. One gram of soil sample was suspended in 9 ml of sterile saline (0.8%) and vortexed. Further dilutions were made from this suspension. For total bacterial count, 100 μl of suspension was spread plated on nutrient agar, and the total number of colonies was counted after incubation at 28 °C for 24 h. The samples were also tested for the effect on nitrogen fixers, siderophore producers, and phosphate solubilizers by spread plating the soil suspension on Jensen’s Medium, Chrome Azurol nutrient agar medium, and Pikovskaya’s medium, respectively. Dewy bacterial colonies on Jensen’s Medium were counted as nitrogen fixers, colonies forming orange-colored zone on chrome azurol agar medium were counted as siderophore producers, and clear zone forming colonies on Pikovskaya’s agar were counted as phosphate solubilizers. The diversity of the culturable bacteria was calculated according to Shannon–Weaver’s diversity indices (*H*′) (Magurran 1988).

The rhizospheric soil samples collected after 40 days of treatment were used for DNA extraction. Total genomic DNA was extracted from soil samples (0.5 g dry equivalent weight) with the Fast DNA SPIN kit (MP biomedical) for soil. Prior to amplification, extracted DNA was visualized on 0.8% ethidium bromide-stained agarose gels via electrophoresis.

PCR was performed with the thermal cycler (Biometra) using universal primers for bacteria, MF341 GC and MR 907 (Muyzer et al. 1993, 1995) which amplify the variable V3 region of 16S rDNA (MF341 GC-^5′^CGC CCG CCG CGC CCC GTC CCG CCC CCG CCC GCC TAC GGG AGG ^3′^and MR 907-^5′^CCG TCA ATT CMT TTG AGT TT ^3′^). Amplification was performed using an initial denaturation at 94 °C for 5 min, followed by 33 cycles of 20 s at 94 °C, 20 s at 57 °C, and for 30 s at 72 °C, with a final extension of 5 min at 72 °C. PCR products from each sample were run on a 9% polyacrylamide gel using an SCIE-PLAS DGGE assembly (Harvard Bioscience, Cambridge). The gel had a denaturing gradient ranging from 30 to 60% (where the 100% denaturant contains 7 M urea and 40% (vol/vol) formamide). The gel was run in 1.0× Tris–acetate–EDTA buffer at 200 V, 60 °C for 30 min, followed by 80 V, 60 °C for 17 h. Then, the gel was stained with SYBR Gold Dye (5.0 µl in 50 ml distilled water) for 30 min.

The DGGE gel was analyzed by 0–1 patterns, and a binary matrix was generated for the bands in the gel, recorded as 1 (present) and 0 (absent). Jaccard similarity coefficient was used to estimate genetic distances between lines. Simplified representation of genetic distances b/w lines was obtained by UPGMA (unweighted pair group method with arithmetic mean) and represented by a dendrogram.

### Statistical analysis

All statistical analyses were performed using STPR software. One-way ANOVA was conducted for plant growth parameters, soil bacterial count and diversity index of *Brassica,* with concentration of AgNPs being the only variable, while two-way ANOVA was used for bacterial counts and diversity index of wheat, cowpea and soil without plant, with number of days after treatment and concentration of AgNPs as the source of variation.

## Results and discussion

### Effect of silver nanoparticle treatment on the growth profile of plants

**Fig. 1:**
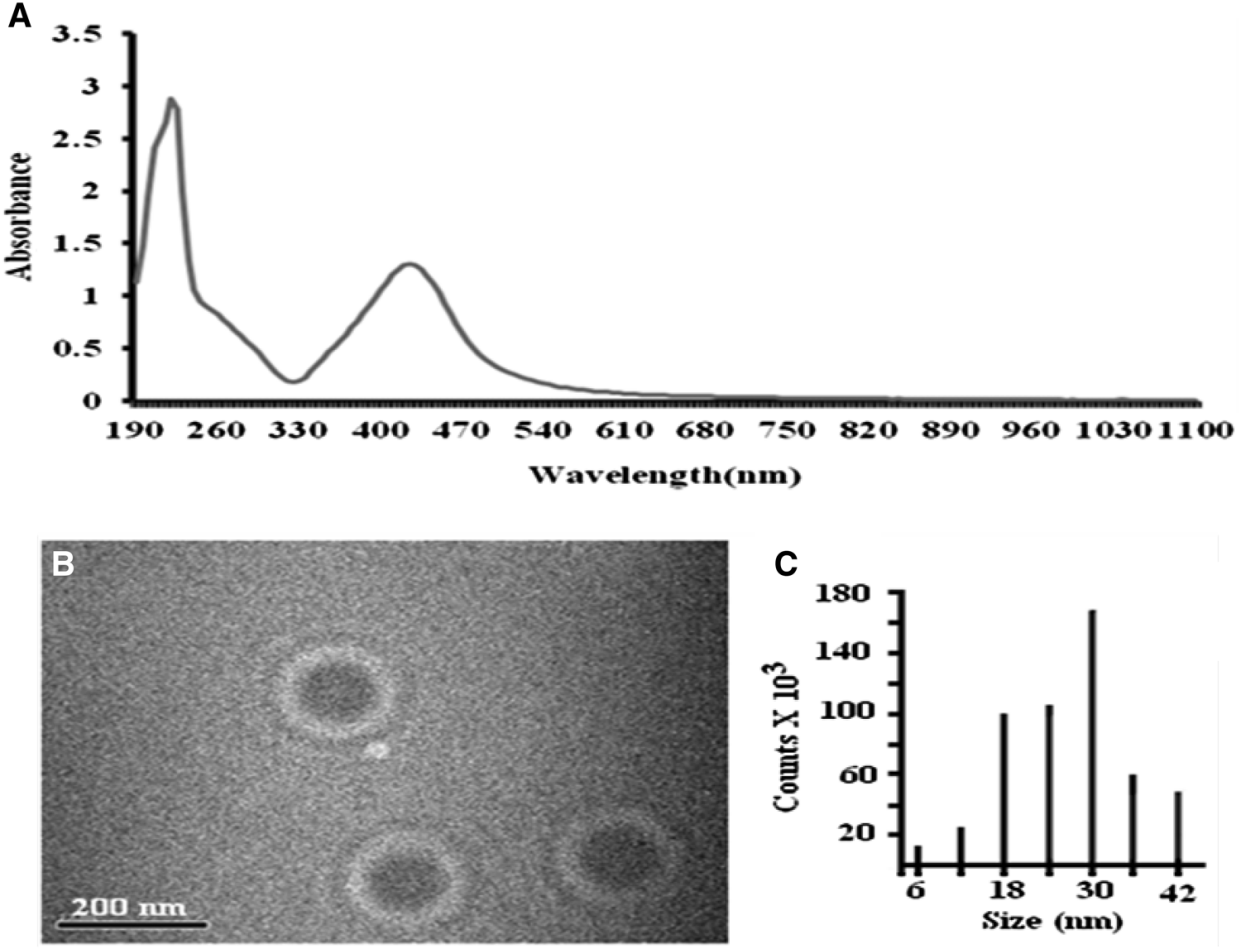
Characterization of silver nanoparticles. **a** UV–VIS spectra in water. **b** Morphology. **c** Particle size distribution

**Fig. 2:**
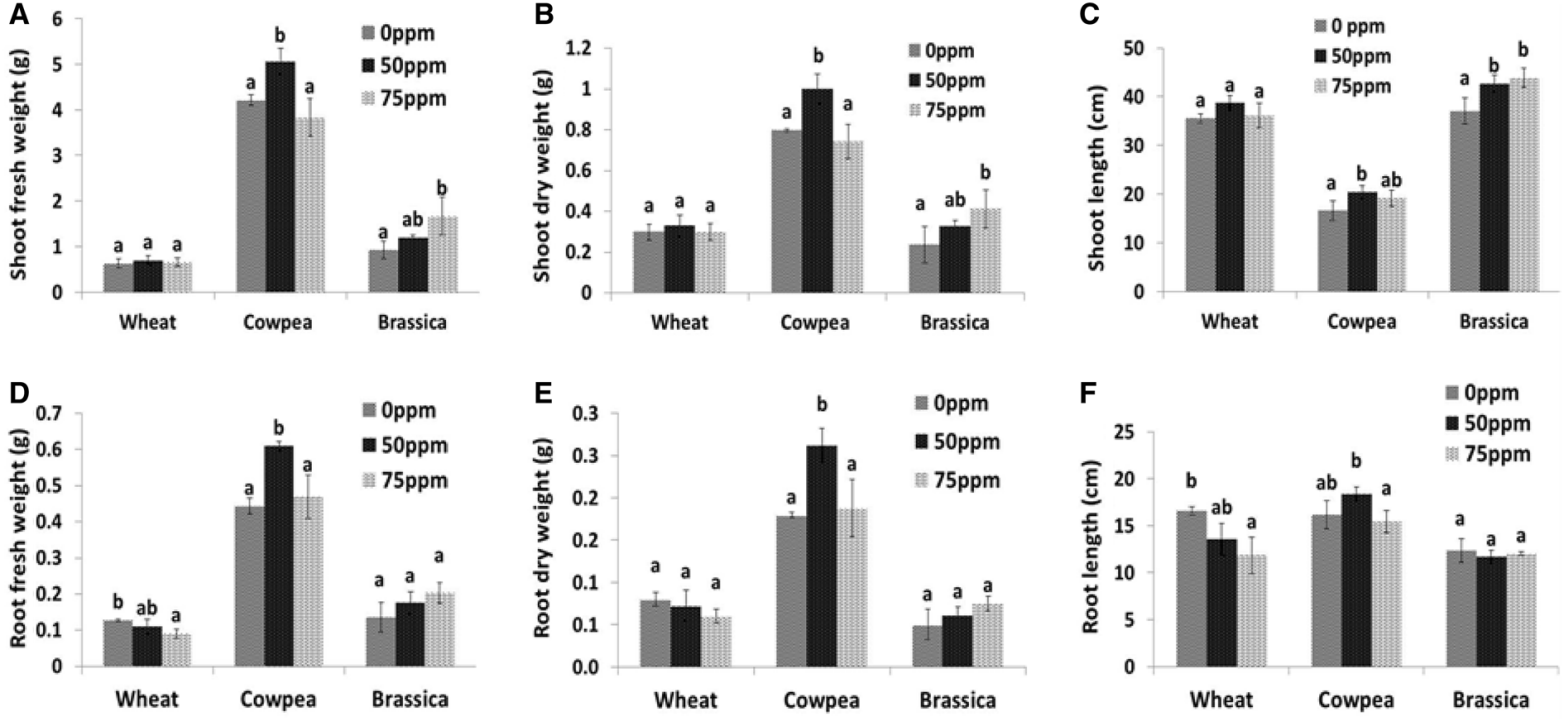
(**a**–**f**) Effect of three different concentrations (0, 50, 75 ppm) of silver nanoparticles on different growth parameters of wheat, cowpea, and *Brassica*

**Fig. 3:**
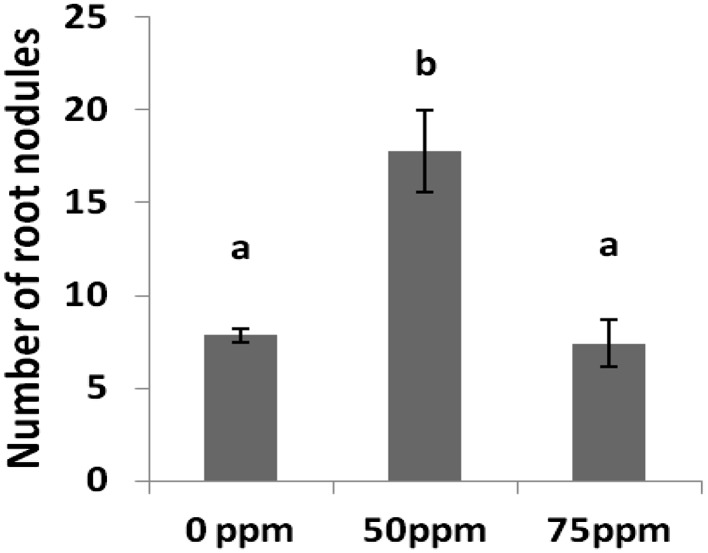
Effect of three different concentrations (0, 50, 75 ppm) of silver nanoparticles on the number of root nodules formed in cowpea

The synthesized AgNPs were characterized by transmission electron microscopy and UV–Vis spectroscopy. Their size ranged between 35 and 40 nm (Fig. 1). The effect of nanoparticles varied from one plant species to another; in wheat, no significant effect of AgNPs was observed on growth parameters, with the exception of root fresh weight and root length, which showed a negative response at 75 ppm treatment, while in cowpea and *Brassica*, a positive response was observed toward AgNPs (Fig. 2). But, the concentration of AgNPs responsible for the observed effects was different for both cowpea and *Brassica*; in cowpea, 50 ppm concentration resulted in growth promotion and increased root nodulation (Fig. 3), whereas in *Brassica* 75 ppm concentration resulted in improved shoot parameters (non-significant effect on root parameters was observed). The exact reasons behind the differential sensitivity of different plants toward NPs remain unknown to this date (Ma et al. 2010; Anjum et al. 2013). Yin et al. (2012) also highlighted the differential susceptibility of 11 different wetland plant species toward AgNPs treatment. Copper NPs were shown to be toxic to two crop species, mung bean (*Phaseolus radiatus*) and wheat (*Triticum aestivum*), as demonstrated by the reduced seedling growth rate (Lee et al. 2008). Nevertheless, Seif et al. (2011) reported an increase in plant height of *Borago* on the application of AgNPs. Regarding the reason for the growth promotion, Arora et al. (2012), in their study, suggested that the changes in the growth profile of *Brassica* seedlings on exposure to gold NPs might be because of the interference of the latter in plant hormone action. Interestingly, Sharma et al. (2012) reported that the AgNPs treatment improved the growth by modulating the antioxidant status of 7-day-old *Brassica* seedlings under in vitro conditions, also as reported by Karimi et al. (2012) AgNPs application did not reduce germinability of wheat seed. Similar to our findings, Sillen et al. (2015) reported that on applying AgNPs to soil, maize plant biomass was significantly enhanced. Increased nodulation was observed in cowpea in case of 50 ppm treatment; however, the exact mechanism behind this cannot be explained on the basis of the current study. Although it can be speculated that since root exudates play an important role in plant microbe interaction (Bais et al. 2006), any change that occurred in root exudation pattern on exposure to this concentration of AgNPs might have been better perceived by the nitrogen-fixing bacteria resulting in enhanced nodulation.

### Evaluation of cultural bacterial diversity of soil

In soil (without plants), total bacterial count improved in 50 ppm treatment. Nitrogen fixers were found to be sensitive toward 75 ppm treatment, while phosphate solubilizers count improved in both 50 ppm and 75 ppm treatment (Table 1). The Shannon diversity index of the total bacterial population and phosphate solubilizers improved after the AgNPs application. However, the diversity of nitrogen fixers was adversely affected (Table 2).

**Table 1:** Effect of silver nanoparticles on the bacterial count (log_10_ cfu) of soil (without plants)

	Total bacteria			N fixers*			P solubilizers^#^		
	20 DAT	40 DAT	Mean	20 DAT	40 DAT	Mean	20 DAT	40 DAT	Mean
0 ppm	2.266^ab^	2.264^a^	2.265^a^	0.667^b^	0.667^b^	0.667^b^	0.201^ab^	0.100^a^	0.151^a^
50 ppm	2.328^c^	2.290^b^	2.309^b^	0.693^b^	0.619^ab^	0.656^b^	0.418^b^	0.593^bc^	0.506^b^
75 ppm	2.283^b^	2.262^a^	2.272^a^	0.460^a^	0.551^ab^	0.506^a^	0.360^b^	0.742^c^	0.551^b^
Mean	2.292^b^	2.272^a^		0.607^a^	0.612^a^		0.326^a^	0.478^b^	
	cd at 5%			cd at 5%			cd at 5%		
Time interval (*T*)	0.011			0.115			0.138		
Concentration (*C*)	0.014			0.141			0.169		
*T* × *C*	0.020			0.200			0.239		

Values are the means of three replicates

Means sharing different alphabets “a”–“c” differ significantly from each other at *p* < 0.05

* Nitrogen fixers

^#^ Phosphate solubilizers

**Table 2:** Effect of silver nanoparticles on Shannon bacterial diversity index of soil (without plants)

Treatments	Total bacteria			N fixers			P solubilizers		
	20 DAT	40 DAT	Mean	20 DAT	40 DAT	Mean	20 DAT	40 DAT	Mean
0 ppm	1.525^b^	1.556^c^	1.541^a^	0.093^b^	0.093^b^	0.093^b^	0.042^a^	0.035^a^	0.039^a^
50 ppm	1.445^a^	1.625^d^	1.535^b^	0.088^a^	0.084^a^	0.086^a^	0.055^a^	0.079^b^	0.067^b^
75 ppm	1.496^b^	1.603^d^	1.549^a^	0.064^a^	0.078^a^	0.071^a^	0.054^a^	0.107^c^	0.080^b^
Mean	1.489^a^	1.595^b^		0.082^a^	0.085^a^		0.050^a^	0.074^b^	
	cd at 5%			cd at 5%			cd at 5%		
Time interval (*T*)	0.019			0.015			0.015		
Concentration (*C*)	0.024			0.019			0.018		
*T* × *C*	0.034			0.026			0.025		

Values are the means of three replicates

Means sharing different alphabets “a”–“d” differ significantly from each other at *p* < 0.05

In cowpea, the total bacterial count declined with increasing AgNPs concentration. The count of nitrogen fixers and siderophore producers improved in 50 ppm treatment, while 75 ppm inhibited their growth. Phosphate solubilizers were insensitive to the treatments in terms of count as well as diversity. In terms of diversity, 75 ppm treatment was inhibitory to total bacteria, nitrogen fixers, and siderophore producers (Tables 3, 4).

**Table 3:** Effect of silver nanoparticles on bacterial count (log_10_ cfu) of cowpea

Treatments	Total bacteria			N fixers			P solubilizers			Siderophore producers		
	20 DAT	40 DAT	Mean	20 DAT	40 DAT	Mean	20 DAT	40 DAT	Mean	20 DAT	40 DAT	Mean
0 ppm	2.277^b^	2.332^c^	2.305^c^	1.629^c^	1.531^b^	1.580^b^	0.519^a^	0.418^a^	0.469^a^	1.187^c^	1.375^c^	1.281^b^
50 ppm	2.282^b^	2.263^b^	2.272^b^	1.774^d^	1.669^c^	1.721^c^	0.502^a^	0.360^a^	0.431^a^	1.441^c^	1.789^d^	1.615^b^
75 ppm	2.208^a^	2.258^b^	2.233^a^	1.514^b^	1.412^a^	1.463^a^	0.492^a^	0.519^a^	0.506^a^	0.519^b^	0.201^a^	0.360^b^
Mean	2.256^a^	2.284^b^		1.639^b^	1.537^a^		0.504^a^	0.432^a^		1.049^a^	1.122^b^	
	cd at 5%			cd at 5%			cd at 5%			cd at 5%		
Time interval (*T*)	0.019			0.046			0.133			0.153		
Concentration (*C*)	0.023			0.057			0.163			0.188		
*T* × *C*	0.033			0.080			0.230			0.266		

Values are the means of three replicates

Means sharing different alphabets “a”–“c” differ significantly from each other at *p* < 0.05

**Table 4:** Effect of silver nanoparticles on Shannon bacterial diversity index of cowpea

Treatments	Total bacteria			N fixers			P solubilizers			Siderophore producers		
	20 DAT	40 DAT	Mean	20 DAT	40 DAT	Mean	20 DAT	40 DAT	Mean	20 DAT	40 DAT	Mean
0 ppm	1.596^c^	1.390^b^	1.493^b^	0.065^b^	0.060^b^	0.062^a^	0.071^a^	0.060^a^	0.065^a^	0.00004^a^	0.00013^a^	0.00008^a^
50 ppm	1.606^c^	1.398^b^	1.502^b^	0.066^b^	0.055^b^	0.061^a^	0.063^a^	0.055^a^	0.059^a^	0.00004^a^	0.00473^b^	0.00238^b^
75 ppm	1.602^c^	1.310^a^	1.456^a^	0.062^b^	0.019^a^	0.041^a^	0.080^a^	0.073^a^	0.077^a^	0.00002^a^	0.00001^a^	0.00002^a^
Mean	1.601^b^	1.366^a^		0.065^b^	0.045^a^		0.071^a^	0.063^a^		0.00003^a^	0.00162^b^	
	cd at 5%			cd at 5%			cd at 5%			cd at 5%		
Time interval (*T*)	0.023			0.018			0.017			0.0014		
Concentration (*C*)	0.028			0.022			0.021			0.0017		
*T* × *C*	0.039			0.031			0.029			0.0024		

Values are the means of three replicates

Means sharing different alphabets “a”–“c” differ significantly from each other at *p* < 0.05

In wheat, total bacterial count showed reduction in 50 and 75 ppm treatments. Nitrogen fixers and siderophore producers were sensitive toward 75 ppm treatment; however, siderophore producer count significantly improved in the 50 ppm treatment and phosphate solubilizer colony count was reduced in 75 ppm treatment (Table 5). An increase in diversity index of total bacterial population was observed in the 50 ppm treatment, whereas the diversity of nitrogen fixers decreased in the 75 ppm treatment. On the other hand, phosphate solubilizers were not affected in terms of diversity (Table 6).

**Table 5:** Effect of silver nanoparticles on bacterial count (log_10_ cfu) of wheat

Treatments	Total bacteria			N fixers			P solubilizers			Siderophore producers		
	20 DAT	40 DAT	Mean	20 DAT	40 DAT	Mean	20 DAT	40 DAT	Mean	20 DAT	40 DAT	Mean
0 ppm	2.299^c^	2.282^c^	2.290^c^	0.920^c^	0.519^b^	0.719^b^	0.360^ab^	0.560^b^	0.460^b^	0.981^c^	0.933^b^	0.957^b^
50 ppm	2.259^bc^	2.271^bc^	2.265^b^	1.075^c^	0.418^ab^	0.747^b^	0.551^b^	0.534^b^	0.543^b^	1.285^c^	1.308^d^	1.296^c^
75 ppm	2.142^a^	2.248^b^	2.195^a^	0.534^b^	0.259^a^	0.397^a^	0.100^a^	0.159^a^	0.130^a^	0.502^b^	0.201^a^	0.351^a^
Mean	2.233^a^	2.267^b^		0.843^b^	0.399^a^		0.337^a^	0.418^a^		0.923^a^	0.814^a^	
	cd at 5%			cd at 5%			cd at 5%			cd at 5%		
Time interval (*T*)	0.017			0.147			0.178			0.114		
Concentration (*C*)	0.021			0.180			0.219			0.140		
*T* × *C*	0.029			0.255			0.309			0.198		

Values are the means of three replicates

Means sharing different alphabets “a”–“c” differ significantly from each other at *p* < 0.05

**Table 6:** Effect of silver nanoparticles on Shannon bacterial diversity index of wheat

	Total Bacteria			N-Fixers			P-solubilizers			Siderophore Producers		
Treatments	20 DAT	40 DAT	Mean	20 DAT	40 DAT	Mean	20 DAT	40 DAT	Mean	20 DAT	40 DAT	Mean
0 ppm	1.443^a^	1.589^d^	1.516^a^	0.133^c^	0.070^a^	0.062^b^	0.052^a^	0.076^a^	0.064^a^	0.00006^b^	0.00013^c^	0.00008^a^
50 ppm	1.470^b^	1.609^d^	1.540^b^	0.179^d^	0.061^a^	0.061^b^	0.078^a^	0.076^a^	0.077^a^	0.00023^c^	0.00473^d^	0.00248^b^
75 ppm	1.485^b^	1.525^c^	1.505^a^	0.095^b^	0.050^a^	0.041^a^	0.052^a^	0.032^a^	0.042^a^	0.00004^a^	0.00004^a^	0.00002^a^
Mean	1.466^a^	1.574^b^		0.136^b^	0.060^a^		0.061^a^	0.061^a^		0.00011^a^	0.00162^b^	

		cd at 5%			cd at 5%			cd at 5%			cd at 5%	
Time Interval(T)		0.015			0.019			0.021			0.00002	
Concentration(C)		0.018			0.023			0.026			0.00003	
T×C		0.025			0.031			0.036			0.00004	

Values are the means of three replicates

Means sharing different alphabets “a”–“d” differ significantly from each other at *p* < 0.05

In *Brassica*, the total bacterial count was higher in 50 ppm treatment, while a slight reduction was recorded in the 75 ppm treatment. Nitrogen fixers’ count also decreased in the 75 ppm treatment. However, the treatments had non-significant effect on phosphate solubilizers (Table 7). Total bacterial diversity increased in the 50 ppm treatment and decreased in the 75 ppm treatment (Table 8).

**Table 7:** Effect of silver nanoparticles on bacterial count (log_10_ cfu) of *Brassica*

Treatments	Total bacteria	N fixers	P solubilizers
	20 days	20 days	20 days
0 ppm	2.281^b^	0.981^ab^	0.418^a^
50 ppm	2.330^c^	1.078^b^	0.551^a^
75 ppm	2.199^a^	0.937^a^	0.593^a^
cd at 5%	0.036	0.098	0.228

Values are the means of three replicates

Means sharing different alphabets “a”, “b” differ significantly from each other at *p* < 0.05

**Table 8:** Effect of silver nanoparticles on Shannon Bacterial Diversity Index of *Brassica*

Treatments	Total Bacteria	N-Fixers	P solubilizer
	**20 days**	**20 days**	**20 days**
0 ppm	1.232^ab^	0.151^a^	0.059^a^
50 ppm	1.260^b^	0.161^a^	0.074^a^
75 ppm	1.164^a^	0.159^a^	0.068^a^
**cd at 5%**	0.078	0.029	0.035

Values are the means of three replicates

Means sharing different alphabets “a”, “b” differ significantly from each other at *p* < 0.05

Although no clear-cut toxicity of NPs was observed toward the soil bacterial community, in some samples 75 ppm concentration was inhibitory. On the other hand, 50 ppm concentration enhanced the bacterial count in some cases. Thus, it can be concluded that the impact of AgNPs is concentration dependent.

### PCR-DGGE analysis using universal bacterial primer

**Fig. 4:**
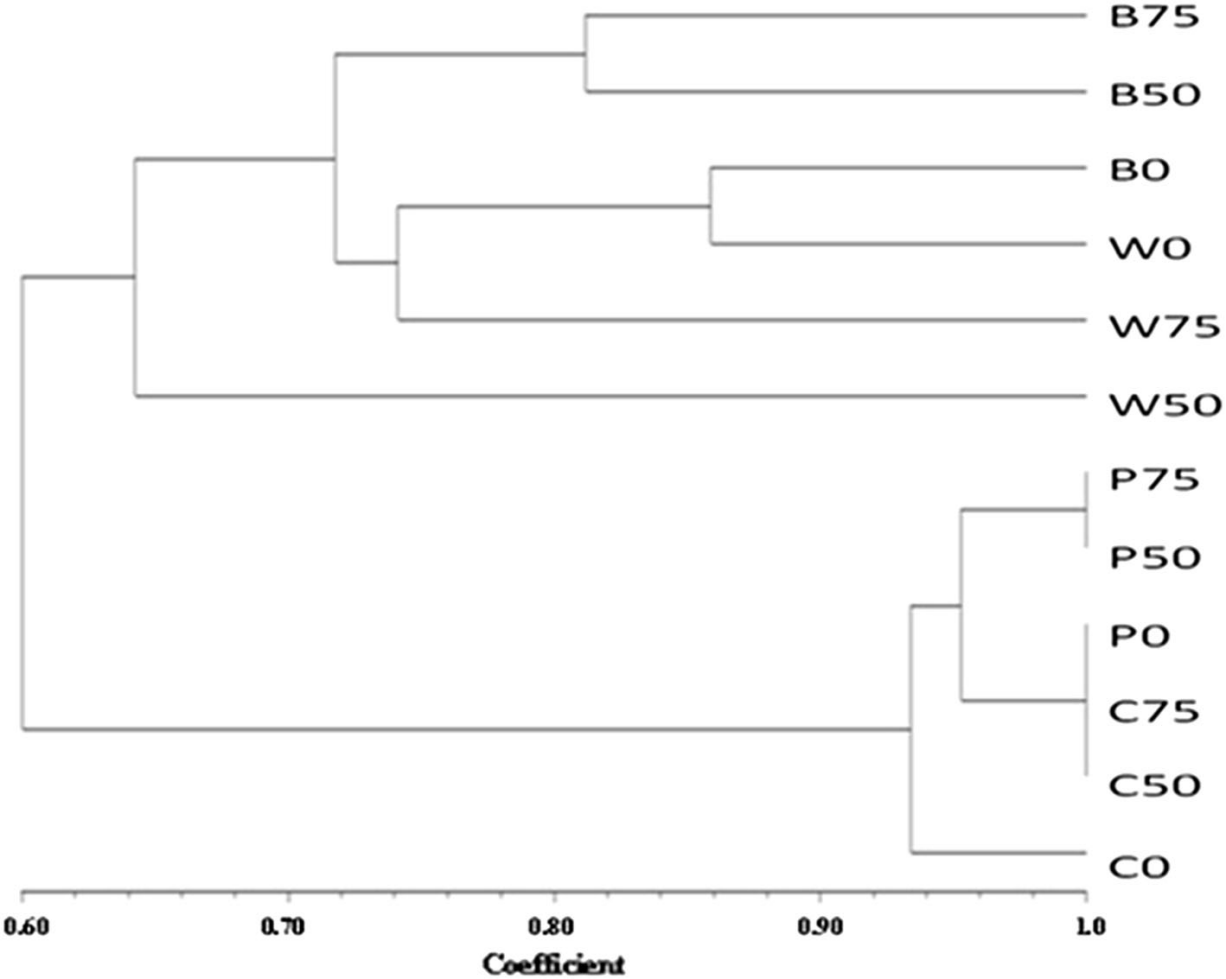
Assessment of shift in bacterial diversity in soil samples by the dendrogram generated on the basis of banding pattern obtained from denaturing gradient gel electrophoresis (P, soil without plants; C, cowpea; W, wheat; B, *Brassica*; as suffix 0 = 0 ppm, 50 = 50 ppm, 75 = 75 ppm

On the basis of the obtained banding pattern, it was found that in soil samples (without plants), 0 ppm-treated soil was 95.5% similar to both 50 and 75 ppm-treated soil in terms of bacterial diversity, while in 50 and 75 ppm no effect was visible. Among the soil samples of cowpea, the bacterial diversities of 50 and 75 ppm-treated soil samples were 100% similar to each other, and both of them were 93% similar to the diversity of 0 ppm-treated soil samples. In case of wheat, application of AgNPs resulted in much differences in diversity among treatments, viz., 74% between 0 and 75 ppm, while 50 ppm treatment showed 63% similarity to both 0 and 75 ppm treatments. In *Brassica*, 50 ppm-treated samples were 81.5% similar to 75 ppm-treated samples in diversity, while 0 ppm-treated soil sample showed 72% similarity to both 50 and 75 ppm soil samples (Fig. 4).

It is clear from the results that the impact of AgNPs on soil bacterial diversity is not only concentration dependent, but also varies with the plant species grown in that soil. This specificity can be attributed to the different root exudation patterns of different plant species. The organic acids released by the plants as root exudates play a key role in influencing the microbial diversity of rhizosphere (Grayston et al. 1998; Bais et al. 2006; Haichar et al. 2008). The composition of root exudates varies from plant to plant and affects the relative abundance of microorganisms in the vicinity of the root (Somers et al. 2004). Furthermore, the application of AgNPs might have caused a change in the root exudation pattern, which, as a result, caused the change in the microbial population within the treatments in the same plant species. These results also suggest that different bacterial groups show different responses toward AgNPs. AgNPs affect the microorganisms by interacting with their cell membrane (Sondi and Salopek-Sondi 2004; Lok et al. 2006). Since the membrane properties differ among microbial groups, it could be the possible reason behind the differential behavior pattern of different bacterial groups toward silver nanoparticles (Gavanji 2013). Also, Anjum et al. (2013) and Dimkpa (2014) stated that the antimicrobial properties of AgNPs can get altered when released in soil due to the undergoing complex array of biotic and abiotic processes, for example, pore water harbors a range of electrolytes that increase the aggregation of AgNPs in soil, thus reducing its size-dependent toxicity (Lee et al. 2012). The dose-dependent effect of AgNPs on soil microbial community was also reported by Chunjaturas et al. (2014). Sillen et al. (2015) in their study suggested a link in increased biomass in maize only when there was a change in soil bacterial community, indicating that the microbial population was altered in a way to promote plant growth.

## Conclusion

From the present study, it can be concluded that plant, microbes, and AgNPs interaction is very complex and, by optimizing the AgNPs concentration, plant growth promotion can be achieved without causing harm to the environment. The findings also highlight the need for further studies to ascertain the reason of differential response of different plant species to AgNP treatments to make them commercially successful.

## Electronic supplementary material

Below is the link to the electronic supplementary material.

Supplementary material 1 (DOCX 804 kb)

## Abbreviations

NPs: Nanoparticles
AgNPs: Silver nanoparticles
PCR: Polymerase chain reaction
DGGE: Denaturing gradient gel electrophoresis
EC: Electrical conductivity
DAT: Days after treatment
