# Supplementary material for: Impact assessment of silver nanoparticles on plant growth and soil bacterial diversity
Source: 3 Biotech. 2016 Nov 28;6(2):254. doi: 10.1007/s13205-016-0567-7 (PMC5125160; doi:10.1007/s13205-016-0567-7)
Supplement: Supplementary file 1 — Supplementary material 1 (DOCX 804 kb) [file 13205_2016_567_MOESM1_ESM.docx]

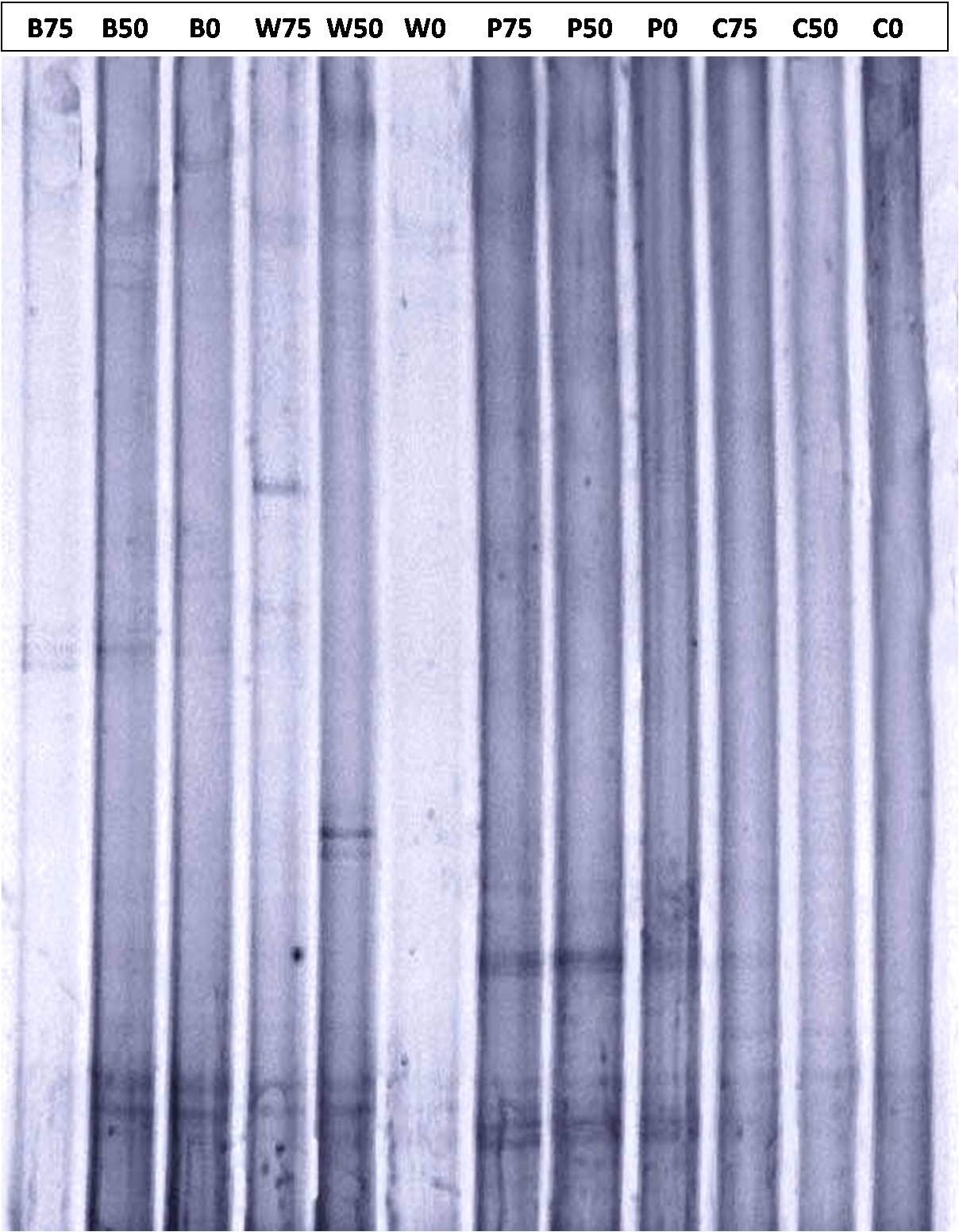


**Fig. 4** Assessment of shift in bacterial diversity in soil samples by the Denaturing Gradient Gel Electrophoresis (P= Soil without plants, C=cowpea, W=wheat, B=*Brassica*; as suffix 0=0 ppm, 50=50 ppm, 75=75 ppm


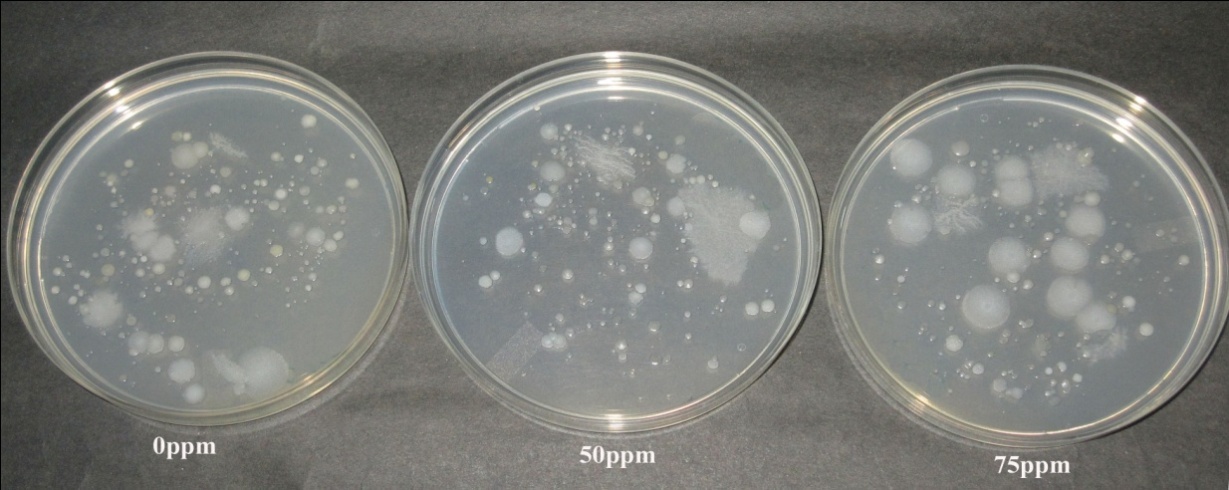
(A)


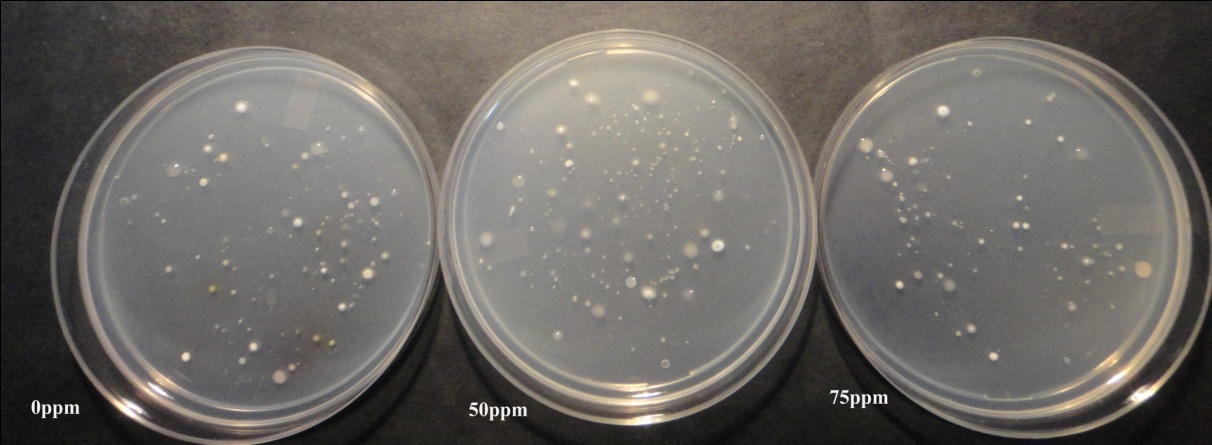
(B)


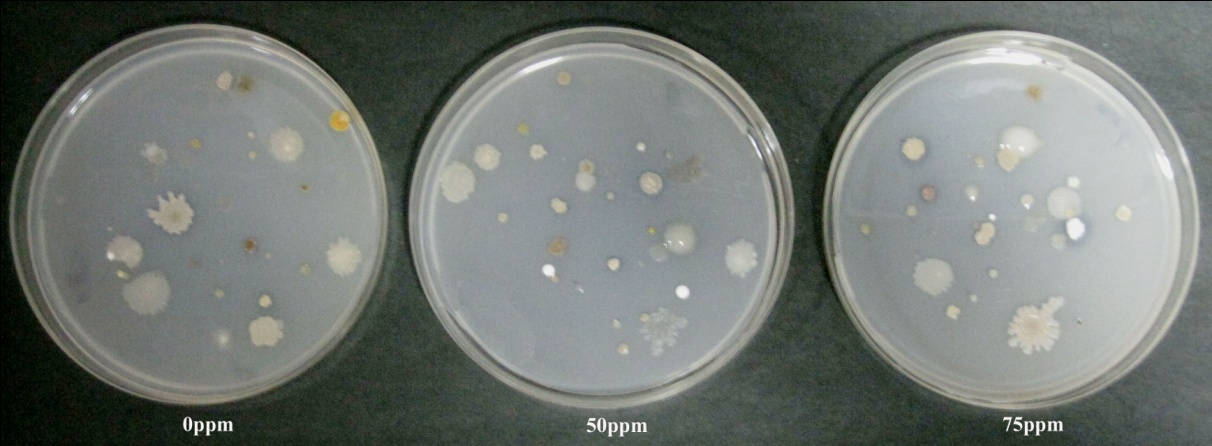
(C)


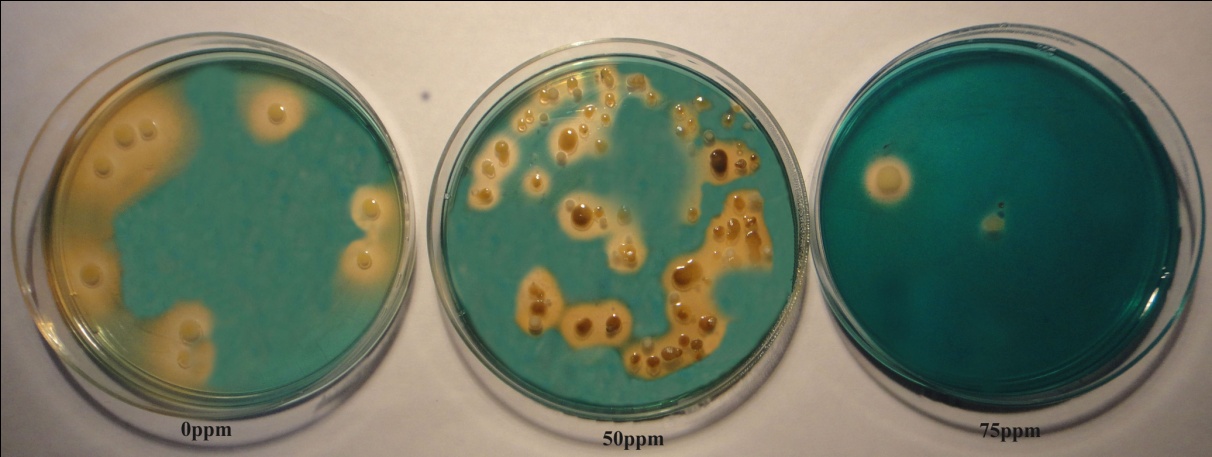
(D)

Fig. 2(A-D) Effect of silver nanoparticles on (A). Total Bacterial population (B) Free living nitrogen fixers (C) Phosphate solubilizers, and (D) Siderophore producers
